# Still and rotating myosin clusters determine cytokinetic ring constriction

**DOI:** 10.1038/ncomms11860

**Published:** 2016-07-01

**Authors:** Viktoria Wollrab, Raghavan Thiagarajan, Anne Wald, Karsten Kruse, Daniel Riveline

**Affiliations:** 1 Laboratory of Cell Physics ISIS/IGBMC, ISIS & icFRC, Université de Strasbourg & CNRS, 8 allée Gaspard Monge, Strasbourg 67000, France; 2 Institut de Génétique et de Biologie Moléculaire et Cellulaire, Illkirch, France; 3 Centre National de la Recherche Scientifique, UMR7104, Illkirch, France; 4 Institut National de la Santé et de la Recherche Médicale, U964, Illkirch, France; 5 Université de Strasbourg, Illkirch, France; 6 Theoretical Physics, Saarland University 66123, Saarbrücken, Germany

## Abstract

The cytokinetic ring is essential for separating daughter cells during division. It consists of actin filaments and myosin motors that are generally assumed to organize as sarcomeres similar to skeletal muscles. However, direct evidence is lacking. Here we show that the internal organization and dynamics of rings are different from sarcomeres and distinct in different cell types. Using micro-cavities to orient rings in single focal planes, we find in mammalian cells a transition from a homogeneous distribution to a periodic pattern of myosin clusters at the onset of constriction. In contrast, in fission yeast, myosin clusters rotate prior to and during constriction. Theoretical analysis indicates that both patterns result from acto-myosin self-organization and reveals differences in the respective stresses. These findings suggest distinct functional roles for rings: contraction in mammalian cells and transport in fission yeast. Thus self-organization under different conditions may be a generic feature for regulating morphogenesis *in vivo*.

## Abstract (web summary)

The cytokinetic ring consists of actin and myosin, but their organisation prior to and during constriction has not been observed. Here the authors observe that mammalian and yeast cells organise their rings differently, with mammalian cells forming a periodic pattern of myosin clusters and yeast rotating myosin clusters during constriction.

## Graphical abstract

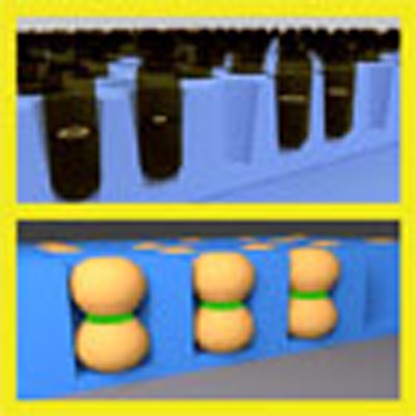

Cytokinesis is the final step of the eukaryotic cell cycle, dividing the cell into two and separating the two nuclei generated during mitosis into the newly formed daughter cells^1^. Animal cells and some fungi use a ring of actin and myosin II to constrict a cleavage furrow at the beginning of cytokinesis^2^,^3^ as shown, for example, with fluorescence microscopy and electron microscopy for *Caenorhabditis elegans*^4^, sea urchin eggs^5^, newts^6^,^7^, mammalian cultured cells^8^ and fission yeast^9^,^10^,^11^,^12^,^13^,^14^.

Concentrations of actin, myosin and actin-associated proteins such as nucleating and crosslinking proteins have been estimated quantitatively for fission yeast^15^ and for *Dictyostelium discoideum*^16^. Understanding cytokinetic ring dynamics has usually been based on muscle acto-myosin with sliding filaments generating forces in a manner analogous to sarcomeres^2^,^17^,^18^. In sea urchin^5^, it was shown that actin volume decreases during constriction, in agreement with proposed filament shortening during closure in fission yeast^12^ where actin and actin-associated proteins decrease in quantity during closure while the total amount of myosin appears to be constant^15^. These results suggest that some ring components, like actin and actin-associated proteins disassemble during constriction, whereas others such as myosins become more concentrated potentially promoting higher forces.

Fission yeast studies suggest that the filament polarities and the distribution of myosin motors are important for mechanical closure^19^. Studies of filament polarity using myosin decoration indicate that actin filaments have a mixed polarity within the cytokinetic ring, although arrays as seen in sarcomeres have not been reported^16^. In addition, the dynamics and the mesoscopic mechanisms of ring closure remain unclear, and this is also true for mammalian cell cytokinetic rings^20^,^21^,^22^,^23^.

In this study, we orient mammalian and fission yeast cells using micro-cavities to image cytokinetic rings in single focal planes^24^,^25^,^26^. We find that the internal organization and dynamics of rings are different from sarcomeres and distinct in the different cell types. In mammalian cells, the periodic pattern of myosin clusters emerges from a homogeneous distribution at the onset of constriction. In contrast, in fission yeast, myosin clusters appear during ring formation^27^. We report that they rotate prior to and during constriction. We use a continuum theory to study acto-myosin dynamics which indicates that both patterns result from acto-myosin self-organization. Furthermore the theoretical analysis reveals differences in the respective stresses generated by the different myosin organizations. We confirm these results with experimental tests. These findings suggest distinct functional roles for rings: contraction in mammalian cells and transport in fission yeast.

## Results

### Orienting cytokinetic rings

**Figure 1:**
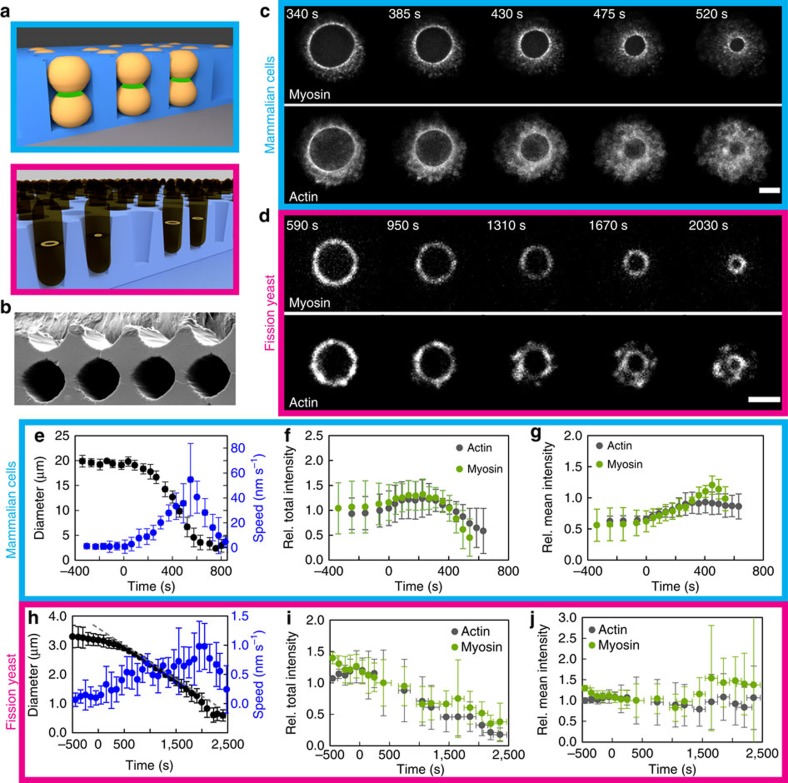
The cytokinetic ring in one plane of focus. (**a**) Cells are oriented in micro-cavities during cytokinesis along the axis of division. (**b**) Electron microscopy image of an array of PDMS micro-cavities. Cavity diameter=25 μm. (**c**,**d**) The cytokinetic ring visualized in mammalian cells (**c**, HeLa) and fission yeast (**d**) by myosin (MHC–GFP in **c**; Rlc1-mCherry in **d**) and actin (Lifeact-mCherry in **c**; CHD–GFP in **d**) (**c**) shows superimposition of five *z*-planes. Scale bar, 5 μm in **c**; 2 μm in **d**. Time zero is the onset of constriction. (**e**,**h**) Ring diameter and the corresponding closure speed as a function of time. (**e**) *N*=14, (**h**) *N*=20. The velocity is computed from individual constriction curves and then averaged. In **h**, the grey dashed line indicates the linear constriction regime. (**f**,**g**) Relative mean (**g**) and total (**f**) intensity of myosin and actin in the cytokinetic ring of mammalian cells. The intensity is normalized for cells at a diameter of 10 μm. *N*=10 for myosin, *N*=6 for actin. (**i**,**j**) Relative mean (**j**) and total (**i**) intensity of myosin and actin in the cytokinetic ring of fission yeast cells. The intensity is normalized for cells at a diameter of 3.1 μm. The intensity measurements were made from individual snapshots of rings for the time period of 300–2,500 s. *N*=241 for both actin and myosin. The intensities before constriction were acquired from time-lapse movies of individual rings (*N*=3). (**e**–**j**) Error bars indicate s.d.

To increase the spatial and temporal resolution, we oriented mammalian cells and fission yeast cytokinetic rings parallel to their plane of observations. We introduced them into microfabricated wells^24^,^25^ prepared by soft lithography (Fig. 1a,b and Supplementary Fig. 1). This allowed the acto-myosin rings to be positioned in a single focal plane (Fig. 1c,d and Supplementary Movies 1 and 2). The ring was visualized by fusion of fluorescent proteins with myosin and actin markers (Methods). Other proteins of both rings were also observed (Supplementary Figs 3 and 8). This orientation allowed us to investigate the inner dynamics of the ring with a time resolution of seconds. We first established that acto-myosin ring constriction in the vertically oriented cells behaved similarly to cells growing horizontally on a microscope slide, after onset of constriction (*t*=0 s, Supplementary Fig. 2). Cells underwent division with timing similar to cytokinesis on flat surfaces. We measured the diameter as a function of time (Fig. 1e,h) during the whole process of cytokinesis, which transforms the mammalian ring from 20 μm diameter to 2 μm within 500 s at 37 °C and the fission yeast ring from 3.5 to 0.3 μm within 40 min at 27 °C. The time courses of the constriction process differed: the velocity of mammalian rings increases monotonously until a diameter of ∼5 μm and stalls at ∼2 μm, whereas the velocity of fission yeast rings increases until ∼3 μm, is then constant until ∼1.5 μm and increases again until complete constriction. To understand the origin of discrepancies between both cell types, we measured total intensities (Fig. 1f,i), and derived the average protein densities (Fig. 1g,j) as an indicator for the generated stress. Changes in myosin and actin densities alone were within 20% only after time 0 s, and do not readily explain the sudden onset of constriction in neither mammalian nor fission yeast rings. This prompted us to take a closer look at the spatial organization of these proteins within the ring at the onset of constriction.

### Still and rotating myosin clusters

**Figure 2:**
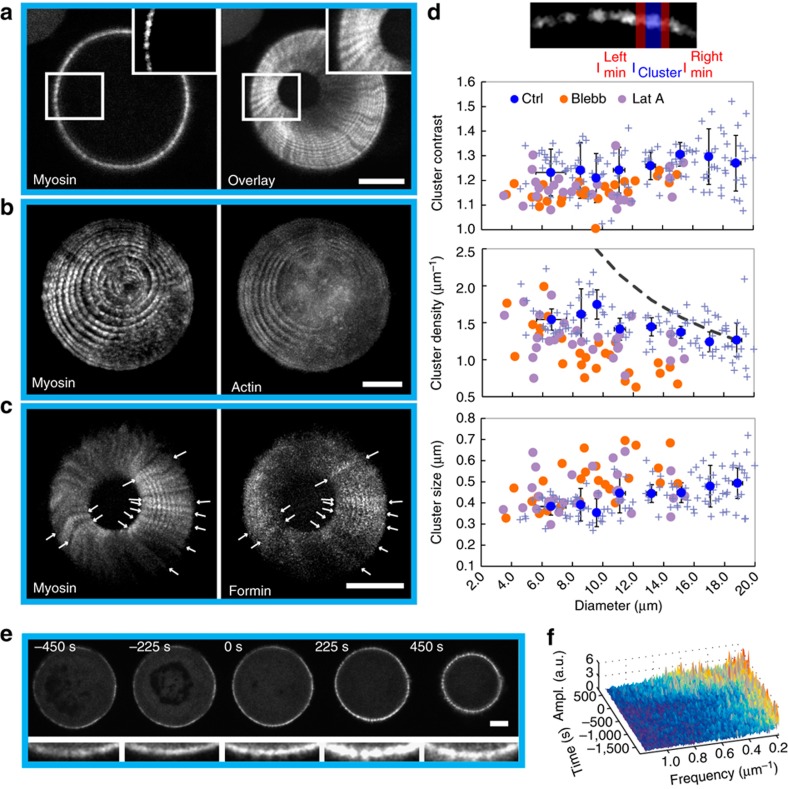
Cytokinetic rings of mammalian cells exhibit regular patterns. (**a**) Myosin is distributed in regular clusters around the perimeter of the cytokinetic ring. Right: overlay of 25 frames, starting from the frame shown on the left. The clusters move radially, though little and collective rotations are visible for larger diameter. Time between images 10 s. (**b**) Overlay of 26 subsequent frames of a closing ring visualized by the fluorescence labelling of myosin and actin. Time between images 45 s, superimpositions of five *z*-planes, plane distance 1.3 μm. (**c**) The cytokinetic ring visualized by the labelling of myosin and mDia2-formin. Formin colocalizes with myosin (arrows). Overlay of 17 frames, time between frames 10 s. (**d**) Characterization of the myosin pattern in the control (blue) and after incubation with the cytoskeleton drugs blebbistatin (orange) and latrunculin A (purple). The cluster contrast stays about constant. The cluster density is increasing while the ring is constricting, but not as much as expected for a constant number of clusters (dashed line). Cluster density of cells treated with the two drugs is reduced. The cluster size decreases as the ring closes. Blue points represent averages, crosses correspond to single data points, error bars indicate the s.d., time-lapses of 11 closing rings were analysed. Each orange (blebbistatin) or purple (latrunculin A) point is obtained from averaging the cluster parameters from a fixed ring (blebbistatin: *N*=25, latrunculin A: *N*=28). (**e**) Time-lapse of a cell before, during and after formation of the cytokinetic ring visualized for myosin, below a zoomed region. Scale bar, 5 μm. (**f**) Fourier spectrum of the intensity profiles from the ring shown in **e**. At *t*=0 s, higher frequency structures appear. Scale bar, 5 μm in **a**–**c**,**e**; *t*=0 s is the onset of constriction.

**Figure 3:**
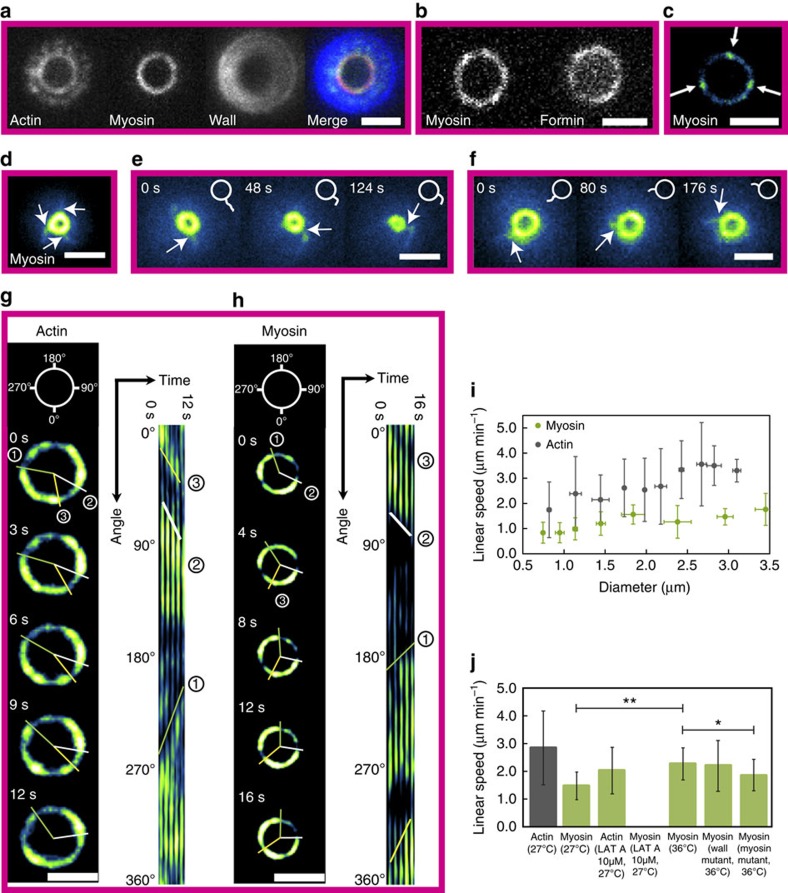
Actin and myosin cluster rotate in fission yeast rings. (**a**) The fission yeast cytokinetic ring visualized by actin (CHD–GFP) myosin (Rlc1-tdTomato) and cell wall (calcofluor) labelling. (**b**) The ring is visualized by myosin and formin (Cdc12–GFP) labelling. Scale bar, 2 μm. (**d**–**f**) Representation of arms extending out of a ring (**d**). Arms attached to the ring (arrows) rotate counter-clockwise (**e**) and clockwise (**f**). The rings are visualized by myosin labelling (Rlc1-mCherry). Scale bar, 2 μm. (**c**,**g**,**h**) Representation of clusters (arrows) in the cytokinetic ring visualized by myosin (Rlc1-mCherry) (**c**). During ring constriction, clusters of actin (CHD–GFP) (**g**) and myosin (Rlc1-tdTomato) (**h**) rotate clockwise and counter-clockwise. The analysis is based on a kymograph representation of the ring after polar transformation. Lines highlight the motion of clusters on the ring and in the kymographs. Scale bar, 2 μm. (**i**) The analysis of cluster motion reveals a decrease in actin cluster speed during constriction. Myosin clusters rotate with a constant speed with decreasing speed towards the constriction completion. Error bars indicate s.d. Each point contains 4–50 measurements, 202 in total for actin and 77 for myosin. (**j**) Comparison of the cluster speed in different conditions and for different proteins. Rings with diameters of >1.5 μm were used. Each mean value contains 13–185 measurements. Error bars indicate s.d. One-way analysis of variance was performed, *P<0.05, **P<0.01.

We first focused on the localization of proteins during mammalian ring closure. Strikingly, the myosin assembles into clusters (Fig. 2a), which are separated by a typical distance of 0.8 μm. In the reference frame of the ring, these clusters persisted at the same location throughout constriction (Fig. 2a and Supplementary Movie 2). The cluster trajectories were mostly straight, although curved trajectories were also observed (Supplementary Fig. 4b). Occasionally neighbouring clusters fused or split (arrows in Supplementary Fig. 4a). Contrasting with actin that showed a less pronounced pattern (Fig. 2b), we detected clusters of the actin nucleator mDia2 (ref. 28), a member of the formin family, co-localizing with myosin clusters (Fig. 2c). In fission yeast, we observed similar clusters for myosin and actin (Fig. 3a–c). Contrary to mammalian clusters, they exhibited motion, rotating typically 0.7 μm clockwise and counter-clockwise in about 20 s within the same rings (Fig. 3g,h, Supplementary Fig. 10a,b and Supplementary Movies 3 and 4), even prior to the onset of constriction. Rotation was further demonstrated through myosin-rich radial extrusions, which extended to the cell periphery during closure (Fig. 3d–f and Supplementary Movie 5). Velocities were measured by polar transformation of the ring to a line followed by kymograph representation, and they were in the μm per min range (Fig. 3g–i). They were larger for actin than for myosin, but both velocities decreased during closure. Despite different mechanical and molecular conditions, cytokinetic rings thus share common motifs in acto-myosin but with different dynamics.

We next asked whether myosins within clusters in both systems were recycling. We measured the turnover time for myosins using Fluorescence recovery after photobleaching (FRAP), and we obtained ∼20 s and ∼1 s for mammalian and fission yeast rings, respectively (Supplementary Figs 6a,b and 10c,d). This is fast compared with the total duration of the closure, 500 s and 40 min, showing fast exchange with cytoplasmic myosin. Despite this myosin dynamics, clusters kept constant features in mammalian rings: the average cluster size (distance between two adjacent fluorescence intensity minima), the average cluster density and the cluster contrast (the ratio of the mean fluorescence intensities of the cluster and the two neighbouring regions) (Fig. 2d). Together, these measurements suggest that myosin clusters involve myosin dynamics while keeping constant their mesoscopic read-outs.

### Myosin clusters and ring constriction

We next wondered whether clusters could be affected by modulating the activity of acto-myosin. Constrictions of mammalian rings in the presence of the myosin inhibitor blebbistatin or the monomeric actin sequestering agent latrunculin A were impaired in both cases while actin and myosin densities remained constant (Supplementary Fig. 5). In addition, the cluster density was reduced, but cluster size was increased compared with normal closure; the cluster contrast was slightly reduced (Fig. 2d). This was associated to stalled constriction for blebbistatin, and opening of rings for latrunculin A. Cluster dynamics was also altered in fission yeast (Fig. 3j): in the presence of 10 μM latrunculin A, velocities of ring constriction were reduced and clusters stopped. In addition, a mutant in myosin (*myo-E1*) was also exhibiting correlations in reduced velocities for ring constriction and clusters. Altogether, these experiments show that acto-myosin activity affects clusters. However, when we blocked the addition of the sugar wall with a wall mutant (*cps1-191*), cluster rotation was preserved with velocities similar to wild-type rings (Fig. 3j). This suggested that rotation is a built-in property of the acto-myosin ring in fission yeast, independently of the wall growth.

While clusters were present in fission yeast prior to ring constriction, mammalian rings already formed exhibited a quasi-homogeneous distribution up to 500 s prior to constriction (Fig. 2e and Supplementary Movie 6) with similar myosin density. Concomitantly with the onset of constriction, this distribution was changed into quasi-periodic clusters as reported above. Fourier transformation of the intensity profile supports the appearance of periodic structures (Fig. 2f). The maximum spatial frequency is consistent with the cluster density measurement. The simultaneous appearance of long lasting clusters during the onset of closure, together with the striking observation that none of the observed rings constricted in the absence of clusters, suggest that self-organization *per se* of myosin rather than an increase in motor density triggers a larger stress inducing constriction.

### Physical model of acto-myosin organization

**Figure 4:**
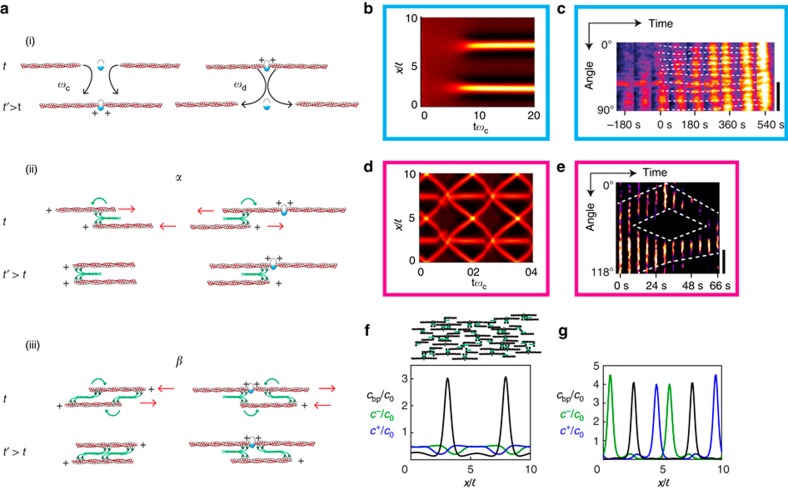
Model for acto-myosin rings. (**a**) Schematic of the model components and their interactions describing the parameters (see text). At each plus-end there is a motor, but only motors interacting with another filament are shown. (**b**,**c**) Kymographs of emerging stationary myosin clusters in the model (**b**) and in mammalian cells (**c**). Myosin density is colour-coded. In **b**, the parameter *α* is increased from a sub- to a supercritical value, the initial distribution was homogenous with a random perturbation. (**d**,**e**) Kymographs of rotating myosin clusters in the model (**d**) and in fission yeast (**e**). Myosin density is colour-coded, and the parameter *α* is constant in **d**. In **c**,**e**, dashed white lines serve as a guide to the eye. (**f**,**g**) Distributions of bipolar filaments (*c*_bp_, black) and polar filaments (*c*^+^, blue, *c*^−^, green) corresponding to *t*=20 *ω*_c_^−1^ of Fig. 4b (**f**) and to *t*=0 of Fig. 4d (**g**). (**f**, top) In alignment, illustration of the F-actin distributions corresponding to two adjacent myosin clusters.

To test the possible mechanisms of pattern formation in cytokinetic rings, we used the physical framework of ref. 29 (Supplementary Note 1). We built the model on generic rules of interactions between parallel and anti-parallel actin filaments through myosin motors (Fig. 4a). Motivated by the observation of coarsening of nodes in fission yeast, we extend the previously developed framework and consider in addition to polar filaments also bipolar structures. In the spirit of a minimal description, we assume that polar filaments of fixed lengths can assemble into bipolar structures at rate *ω*_c_ and disassemble at rate *ω*_d._ Molecular motors induce sliding between parallel and anti-parallel filaments at effective velocities *α* and *β*, respectively. Turnover of actin filaments is taken into account through an effective velocity *v*_to_.

Our analysis revealed that there is a critical value *α*_c_, such that the homogeneous state is unstable against perturbations for *α*>*α*_c_, which only weakly depends on the value of *β*. Then myosin clusters appeared (Fig. 4b) which were stationary in some cases and moving in others, where *ω*_d_ was larger (Fig. 4d). Stationary clusters resembled the pattern observed in mammalian cells: after randomly perturbing a homogenous myosin distribution, the system develops clusters in the myosin distribution separated by a typical distance. These states remain essentially unchanged for simulated times longer than hours and would appear stationary during the course of ring constriction in a living cell. Only after these very long periods would two clusters fuse and further fusion events would take even longer time. Mathematically, the stationary state seems to contain only one cluster. In the case of moving clusters, we always observed pairs of clusters moving in opposite directions co-existing with stationary clusters. These phenomena were visible in kymographs of rings in mammalian cells and in fission yeast (Fig. 4c,e and Supplementary Fig. 9). The apparent discrepancy between the extension of clusters in Fig. 4d and in Fig. 4e may result from the point spread function of the set-up. In addition, the regular pattern of the simulation is modified by molecular noise. Importantly, the mechanism studied by the simulation reproduces co-existing clusters rotating in opposite directions, which is the key feature of myosin in fission yeast rings.

The instability of the homogenous state results from the presence of bipolar filaments. Motors tend to approach the centres of two overlapping bipolar filaments. In the homogenous state, the total force on a bipolar filament vanishes. If this balance is broken by a perturbation, bipolar filaments will accumulate in clusters if their interaction, which is given by the parameter *α*, outcompetes the homogenizing effects by the diffusion term. This mechanism yields a typical distance between clusters of four filament lengths (Supplementary Note 1), which we indeed observe in our calculations (Fig. 4f, top). Polar filaments essentially follow the dynamics of the bipolar filaments. Note, that in steady state, there are still non-vanishing filament fluxes: motors drive bipolar filaments into clusters, while filament assembly and disassembly that we capture by treadmilling, lead to outflux of polar filaments from clusters. The steady state densities show furthermore that the myosin clusters are associated with the bipolar filaments (Fig. 4f, bottom). For the oscillatory state, the densities reveal that stationary clusters are associated with bipolar filaments, whereas mobile clusters are linked with polar filaments (Fig. 4g).

**Figure 5:**
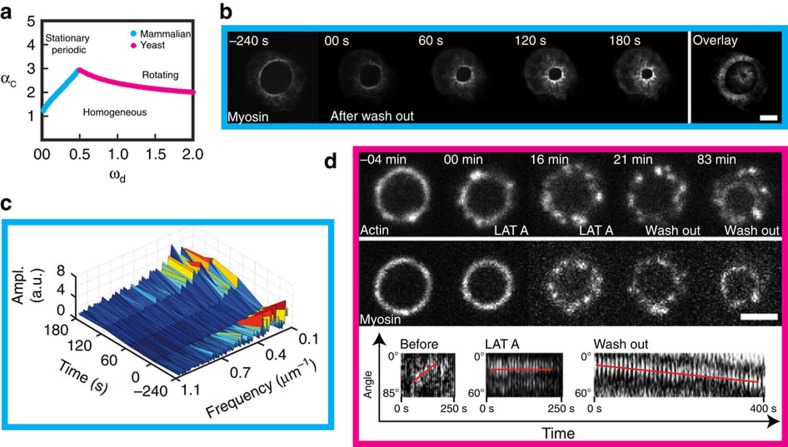
Myosin clusters form through a dynamic instability. (**a**) Critical value of the parameter *α* from a linear stability analysis. Blue: stationary instability; pink: oscillatory instability. (**b**) Mammalian cells before and after 10 min incubation with 100 μM blebbistatin. After drug treatment, the myosin pattern is not present but reappears after wash out. Superimposition of five *z*-planes; scale bar, 5 μm. (**c**) Fourier spectrum of the intensity profiles shown in **b**. Characteristic frequencies are seen before blebbistatin addition and starts to reappear after blebbistatin wash out at 0 s. (**d**) Fission yeast cell before and after 20 min incubation with 10 μM latrunculin A. The ring disassembles, but motion of actin clusters (CHD–GFP) is still visible (see Supplementary Movie 9) while myosin (Rlc1-tdTomato) clusters are still. After wash out, the ring re-forms and constricts again. Scale bar, 2 μm. Polar-transformed kymographs of the ring in myosin (Rlc1-tdTomato) confirm different behaviours of the clusters before and during the incubation of latrunculin A and after wash out.

We then systematically investigated the effect of changes in both parameters *α* and *ω*_d_ (Fig. 5a). Three states were apparent: homogenous distribution, stationary clusters and dynamic clusters co-existing with stationary clusters. Changes of other parameters also affect the system, for example, changes in filament lengths and/or in the velocity can induce transitions from rotating to still clusters and vice versa. Moreover we qualitatively reproduced the changes in cluster contrast and cluster size reported above (Fig. 2d) when changing actin polymerization and myosin activity (Supplementary Note 1).

We wanted to evoke experimentally the transition from homogeneous to patterned states, as well as immobilizing rotating clusters. To effectively move in parameter space, we applied different drugs. For mammalian rings, we reduced myosin activity, which corresponds to a reduction of the parameter *α* in the model, by incubating with 100 μM blebbistatin and found homogenization of myosin. Strikingly, after washing the compound, constriction was re-initiated together with the formation of myosin clusters (Fig. 5b,c and Supplementary Movie 7). This further supports our conclusion that cluster formation triggers constriction. For fission yeast rings, cells were incubated with 10 μM latrunculin A and myosin clusters were stopping with a stalled ring (Fig. 5d and Supplementary Movie 9). After washing the compound, rotation was re-initiated together with ring constriction. These drug experiments illustrate that the inner dynamics of cytokinetic rings is qualitatively captured by our theory.

### Stress and myosin clusters

**Figure 6:**
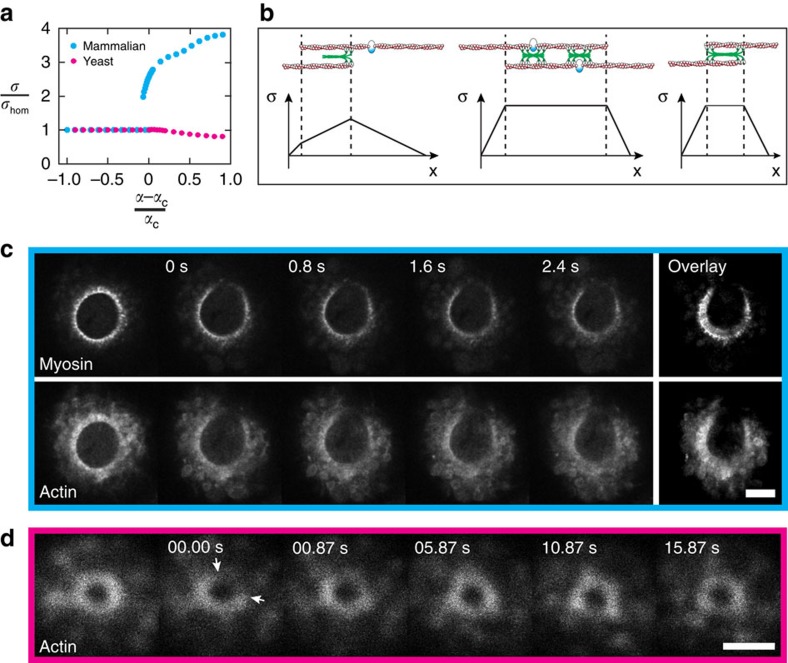
Motor-induced mechanical stresses in contractile rings. (**a**) Calculated stress *σ* as a function of myosin activity *α* undergoing a stationary (blue) or oscillatory (pink) instability. *σ*_hom_ is the stress in homogeneous state. (**b**) Illustration of the stress profiles for pairs of a polar and a bipolar filament (left), two bipolar filaments (middle) and two polar filaments of opposite orientation (right). Stress is drawn to scale. Only motors crosslinking two filaments are shown. (**c**,**d**) Laser ablation of cytokinetic rings in mammalian cells (**c**) and in fission yeast (**d**). (**c**) Ring opens on cutting. Overlay (9 frames, 0.8 s) reveals constriction and radial movement of myosin clusters. Scale bar, 5 μm. (**d**) The ring breaks after cutting (arrows) but is repaired within seconds. Image smoothed with ImageJ. Scale bar, 2 μm.

We next looked for potential functions of such dynamics by calculating the stress associated with still and rotating clusters (Fig. 6a). We evaluated the stress in the ring by summing at each point the stresses in all filaments overlapping with this point^21^,^30^ (Supplementary Note 1 and Fig. 6b). With increasing values of *α*, the stress also increased monotonically. At the value of *α*_c_ for which the homogenous filament distribution becomes unstable and leads to stationary patterns, the sensitivity of the stress to changes in *α* increased dramatically (Fig. 6a). As a consequence, the spontaneous rearrangement of filaments in the ring is accompanied by an increase in the stress generated by the ring that is significantly larger than the increase expected for a homogenous ring with increasing *α* (Fig. 6b). In contrast, the stress changes less than expected for the homogeneous state when the clusters were rotating (Fig. 6a). This difference suggests distinct roles for still and rotating clusters, contraction and transport, respectively, and we turned back to experiments to probe this prediction.

We reasoned that a ring under tension would continue to contract after rupture. We therefore locally ruptured rings by laser ablation (Methods). Indeed, with laser ablation, the mammalian ring opens (Fig. 6c). The remaining bundle further contracted (Supplementary Fig. 7) while myosin clusters moved radially. In contrast, ablated fission yeast rings were healing within seconds without constriction (Fig. 6d). Also, clusters continued to rotate after ablation, suggesting that the remainder of the ring was not affected by ablation. In addition, we acquired a ring with a portion locally detached and ruptured (Supplementary Movie 8). Both new free ends fluctuated over timescales of seconds: this suggests that the ring is not contractile because for a contractile ring the detached parts would retract and straighten. Finally, we also tracked proteins involved in the synthesis of the wall: Bgs4 clusters were also rotating (Supplementary Fig. 9c). These results suggest that the myosin cluster rotation could be related to transport of the wall machinery in fission yeasts rings.

## Discussion

The different functions of the myosin clusters reported above are related to distinct network states and emerge from simple interactions rules between actin and myosin. Consequently, they can play an essential role in a variety of situations where signalling pathways may fail to give satisfactory explanations. These situations notably comprise fission yeast ring formation^27^, *in vitro* rings^31^ and acto-myosin networks^32^, stress fibres in mammalian cells^33^, cortices in *C. elegans*^34^, and cytoplasmic networks in *C. elegans*^35^ and in *Drosophila*^36^.

## Methods

### Microfabrication

Micro-cavities were prepared with standard microfabrication methods (see Supplementary Fig. 1 and Supplementary Methods). Briefly, silicon masters prepared with deep reactive ion etching or photolithography and filters were used to prepare molds^24^. Cavities were prepared by pouring liquid polydimethylsiloxane (PDMS) onto these masters; PDMS was allowed to cure at 65 °C for 4 h, before being unpeeled carefully. These stamps were passivated and then pressed on PDMS-coated coverslips to mold the final micro-cavities. Alternatively passivated stamps were coated with PDMS. After curing, the PDMS layer was bound to coverslips and the stamp unpeeled.

### Experimental chamber preparation

For experiments with mammalian cells, functionalized cavities were used. Cavities are plasma activated and incubated for at least 1 h at room temperature or overnight in the fridge with 20 μg ml^−1^ fibronectin (Sigma). Cavities for experiments with fission yeast are not coated. Prior to observation, HeLa cells were synchronized by mitotic shake off^37^. Cells were inserted in cavities by centrifuging the cell suspension on the cavities three times with 800*g*, each time 2 min (mammalian cells)/5 min (fission yeast). The coverslip with cavities was supported by a custom-made plastic cylinder in a 50 ml vessel. For fission yeast experiments, the centrifuge was preheated to the temperature of the experiment. The coverslip with cavities was removed from the holder and was inserted in a home-made chamber. Removing cells on top of the cavities by gently rinsing of the sample improved the imaging quality. Fresh medium was immediately added to the formed chamber.

We checked that neither cells were rotating within cavities by following external fiducial markers, that is, stationary motifs in cells visualized by phase contrast microscopy. In an alternative set-up shown in Supplementary Fig. 2, coverslips with cells were oriented vertically with respect to the plane of focus. The coverslip was held by two binder clips^38^. The coverslip was patterned with fibronectin (Cytoskeleton) by standard microcontact printing protocols^39^. Mammalian cells were synchronized with the double thymidine block^40^.

### Cell culture

HeLa cells expressing mCherry-Lifeact and MHC–GFP or only MHC–GFP were used (gift from Anthony Hyman, MPI-CBG Dresden). They were cultured in DMEM with 10% FCS, 2 mM L-glutamine and 1% penicillin/streptomycin antibiotics (all Invitrogen). For imaging, we used L-15 medium (Invitrogen) supplemented with 10% foetal calf serum and 2 mM L-glutamine. Fission yeast was grown in exponential phase at 32 °C, and then diluted in YE5S medium (MP Biomedicals). The strains JW1348 and JW1349 (see Supplementary Table 1) were cultured in EMM5S (MP Biomedicals) at 27 °C for 18–24 h before acquisition. Cultures which had reached an optical density of 0.2–0.8 were used for experiments. We visualized entire rings in our set-up with a variety of *Schizosaccharomyces pombe* strains reported in Supplementary Table 1. At the restrictive temperature (36 °C), the temperature sensitive strain PN 4461 was observed for 2–5 h after the temperature shift.

The generic terms ‘myosin' and ‘actin' are used throughout the article for clarity: they refer to MHC and Lifeact for mammalian cells rings, and to Rlc1 and CHD for fission yeast rings, respectively.

### Transfection

The mDia2 plasmid was a gift from Watanabe^28^. We transfected cells with Lipofectamin 2000 (Invitrogen).

### Cytoskeleton drugs and staining

Blebbistatin was used at a concentration of 100 μM and latrunculin A (Sigma) at a concentration of 1.5 μM (mammalian cells)/10 μM (fission yeast). To expose cells immediately with the drugs, the compounds were diluted to the indicated concentration in imaging medium before addition. Medium in which the cells were incubated was removed and the medium with the drug was added. At the end of the incubation time, the drug containing medium was removed and replaced by fresh medium. To characterize the pattern in mammalian cells, cells were incubated for 15 min with blebbistatin (100 μM) or 1.5–3 min with latrunculin A (1.5 μM) and then fixed with 3% paraformaldehyde (PFA, Sigma). For staining, HeLa cells were fixed with 3% PFA (Sigma) and permeabilized with Triton (Sigma). Immunostaining was performed using anti-anillin (gift from M. Glotzer, 1:500) and anti-septin7 (Proteintech, 13818-1-AP, 1:500) primary antibodies.

### Optical set-ups

To acquire cytokinesis in HeLa cells, we used the Leica TCS SP-5-MP or SP-8-MP confocal, upright microscopes equipped with a Leica Application Suite Advanced Fluorescence LAS AF 2.6.3.8173/LAS AF 3.1.2.8785 acquisition system with photomultiplier tube (PMT) and hybrid detectors. We used a × 25 or × 63 HCX IR APO L water objective (0.95 numerical aperture (NA), Leica). Cytokinetic rings in fission yeast cells were acquired with a × 100 HCX PL APO CS oil objective (1.4 NA, Leica) mounted on a spinning disk microscope based on a Leica DMI6000 inverted microscope, equipped with a Yokogawa CSU22 spinning disk unit and Andor iQ 1.9.1 acquisition system. For laser ablation experiment, we used the TCS SP-5-MP (HeLa cells) or SP-8-MP (fission yeast) upright microscopes with an infrared femtosecond pulsed lasers (SP-5: Coherent Ultra, SP-8: Coherent Vision II with precompensation). Rupture in contrast to bleaching was checked by measuring changes in the ring curvature right after ablation. Experiments to measure the constriction were also performed on epifluorescent microscopes (Nikon Eclipse Ti inverted microscope (× 60, oil, 1.40 NA, DIC, Nikon) and Olympus CKX41 inverted microscope (× 100 UPlanFl, oil, 1.30 NA, Olympus, × 100 UPlanSApo, oil, 1.40 NA, Olympus and × 60 PlanApo, oil, 1.45 NA, Olympus). For FRAP experiments on HeLa cells, we used the Leica TCS SP2 AOBS MP based on a Leica DMIRE2 microscope, equipped with a PMT detector and a × 63 HCX PL APO oil (1.4 NA, Leica) objective. Experiments with HeLa cells were performed at 37 °C and with fission yeast at 27 °C if not indicated differently.

### Analysis

We measured the ring diameters with ImageJ. The onset of constriction was set at *t*=0 s. Individual diameter–time plots were aligned at diameter 10 μm for mammalian rings and averaged. The speeds were computed through the changes in diameters over time steps of 100 s for yeast and 45–60 s for mammalian cells. The intensities and intensity profiles were measured with ImageJ. To determine the intensity during closure, we used single images and time-lapse movies (see Supplementary Methods). The time-lapse movies were needed to extract a time information in regimes where the radius does not change over time. The cluster contrast was determined by dividing the intensity of a cluster by the mean intensity of the two neighbouring minima. The cluster density corresponds to the numbers of clusters per perimeter. To analyse the formation of the pattern in the cytokinetic ring in mammalian cells, the intensity profiles were measured with ImageJ and a Fourier Transformation was performed with Matlab. We determined the cluster velocities in fission yeast rings as follows. The rings were transformed to lines (Fig. 3g,h) by a polar transformation (ImageJ) and represented as kymographs. In these kymographs, the cluster can be traced as lines. If the cluster motion could be followed over at least three successive images, we measured their velocities by taking the slopes of their movement. Cluster lifetime and travelling length was also extracted from the kymographs. For better representation, we smoothed images of mammalian cells with ImageJ. For the rings in Figs 2a,c and 3g,h, and Supplementary Movie 4, we doubled the pixel and then smoothed the image. In Fig. 5b, we normalized the stack histogram.

### Statistical analysis

All error bars indicate the s.d. of the mean. For the comparison of cluster velocities in fission yeast cells in Fig. 3j, we considered ring diameters that were equal and larger than 1.5 μm. The statistical significance was tested with one-way analysis of variance and accepted at *P*<0.01 and *P*<0.05. For the comparison of the myosin cluster in mammalian cells in drug-treated and control cells (Supplementary Fig. 5g), we took measurements on rings in the diameter range of 9–12 μm. The statistical significance was tested with Mann–Whitney test and accepted at *P*<0.01 and *P*<0.05.

### Data availabilty

All relevant data are available from the authors.

## Additional information

**How to cite this article:** Wollrab, V. *et al.* Still and rotating myosin clusters determine cytokinetic ring constriction. *Nat. Commun.* 7:11860 doi: 10.1038/ncomms11860 (2016).

## Supplementary Material

Supplementary Information Supplementary Figures 1-12, Supplementary Table 1, Supplementary Note 1, Supplementary Methods and Supplementary References

Supplementary Movie 1 Four rings in focus. Several fission yeast cells imaged simultaneously. Rings are visualized by myosin (Rlc1-mCherry) labelling. Time in hh:mm:ss. Temperature 18°C.

Supplementary Movie 2 Cytokinetic ring constriction of mammalian (HeLa) cell. Full constriction of mammalian cytokinetic ring in both actin (Lifeact-mCherry) and myosin (MHC-GFP). Time in mm:ss.

Supplementary Movie 3 Cytokinetic ring constriction of fission yeast cell. Full constriction of fission yeast cytokinetic ring in both actin (CHD-GFP) and myosin (Rlc1-mCherry). Actin and myosin clusters are visible. Time in mm:ss. Temperature 27°C.

Supplementary Movie 4 Rotating clusters in the cytokinetic ring of fission yeast. Clusters in actin (CHD-GFP) and myosin (Rlc1-mCherry) of different cells. Time in mm:ss. Temperature 27°C.

Supplementary Movie 5 Arm rotations in the cytokinetic ring of fission yeast cell. Myosin (Rlc1-mCherry) arms emerging from the ring of two different fission yeast cells, rotate in clockwise (CW) and counter-clockwise (CCW) directions. Time in mm:ss. Temperature 27°C.

Supplementary Movie 6 Cytokinetic ring formation of mammalian (HeLa) cell. Formation phase of the mammalian cytokinetic ring followed by constriction in both actin (Lifeact-mCherry) and myosin (MHC-GFP). Overlay of 5 z-planes, time in mm:ss.

Supplementary Movie 7 Constriction after blebbistatin wash out in mammalian (HeLa) cell. Myosin pattern of mammalian ring in constriction phase is rescued after blebbistatin is washed out. The ring proceeds to constrict. The ring is visualised by myosin (MHC-GFP) labelling. Overlay of 5 z-planes, time in mm:ss.

Supplementary Movie 8 Local fluctuations of detached parts of the cytokinetic ring in fission yeast. A portion of the ring is spontaneously detached and severed (see arrowhead) : the two new free ends fluctuate while constriction proceeds elsewhere. Myosin is visualized with Rlc1-mCherry. Time in mm:ss. Temperature 27°C.

Supplementary Movie 9 Latrunculin A (10μM) treated fission yeast rings. Motion of actin clusters (CHD-GFP) is still visible, while myosin (Rlc1-tdTomato) clusters are still. Time in mm:ss; 27°C.
