# Supplementary material for: Still and rotating myosin clusters determine cytokinetic ring constriction
Source: Nat Commun. 2016 Jul 1;7:11860. doi: 10.1038/ncomms11860 (PMC4932180; doi:10.1038/ncomms11860)
Supplement: Supplementary Information — Supplementary Figures 1-12, Supplementary Table 1, Supplementary Note 1, Supplementary Methods and Supplementary References [file ncomms11860-s1.pdf]

Supplementary Figures

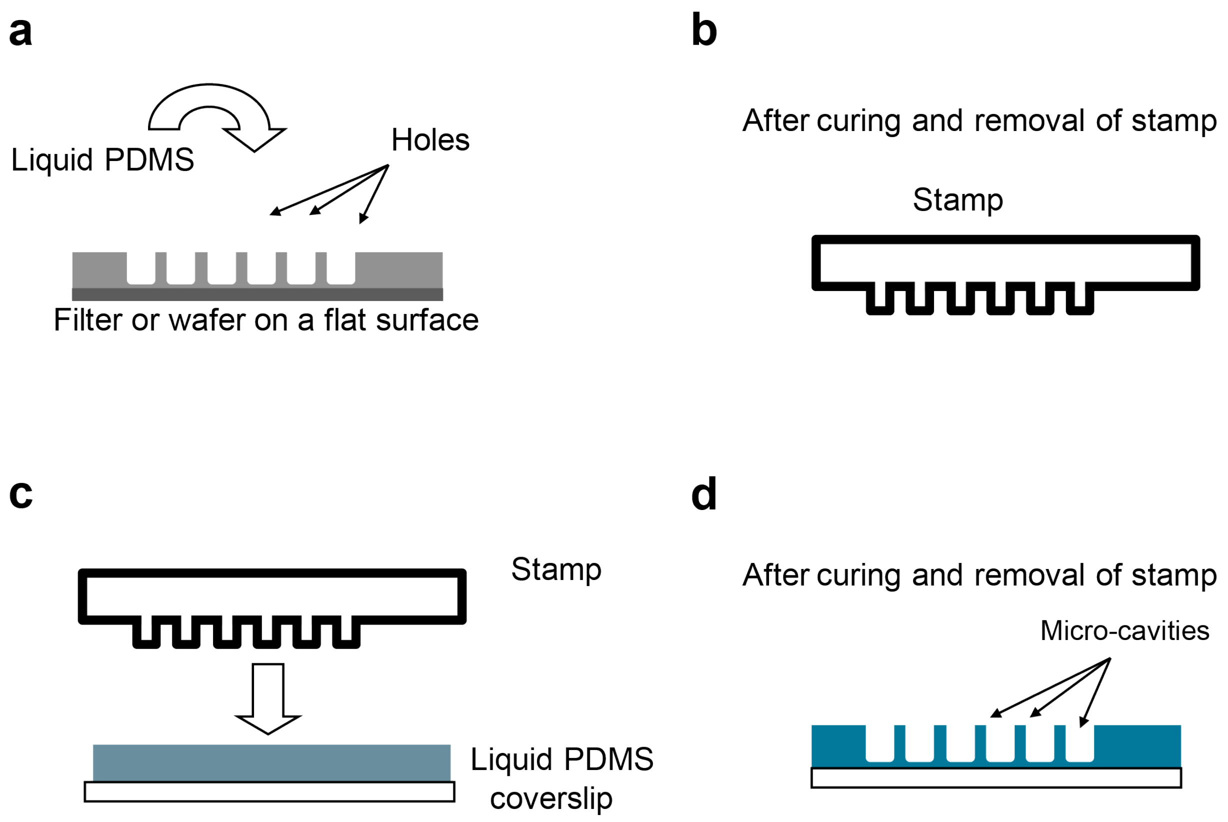

Supplementary Figure 1. Microfabrication of micro-cavities
a, Liquid PDMS is poured on the microstructured surface (filter or wafer). **b,** The PDMS is degased for 30 min, cured for 4h at 65°C. The stamp is cut out and can easily be removed from the surface. **c,** Liquid PDMS is spin coated on plasma activated a glass coverslip (#0). The stamp is silanized and carefully put on the PDMS. **d,** The PDMS is kept for about 1h at room temperature to let gently air bubbles escape from the liquid PDMS. It is then cured for 4 h at 65°C. The stamp is very carefully removed[1].

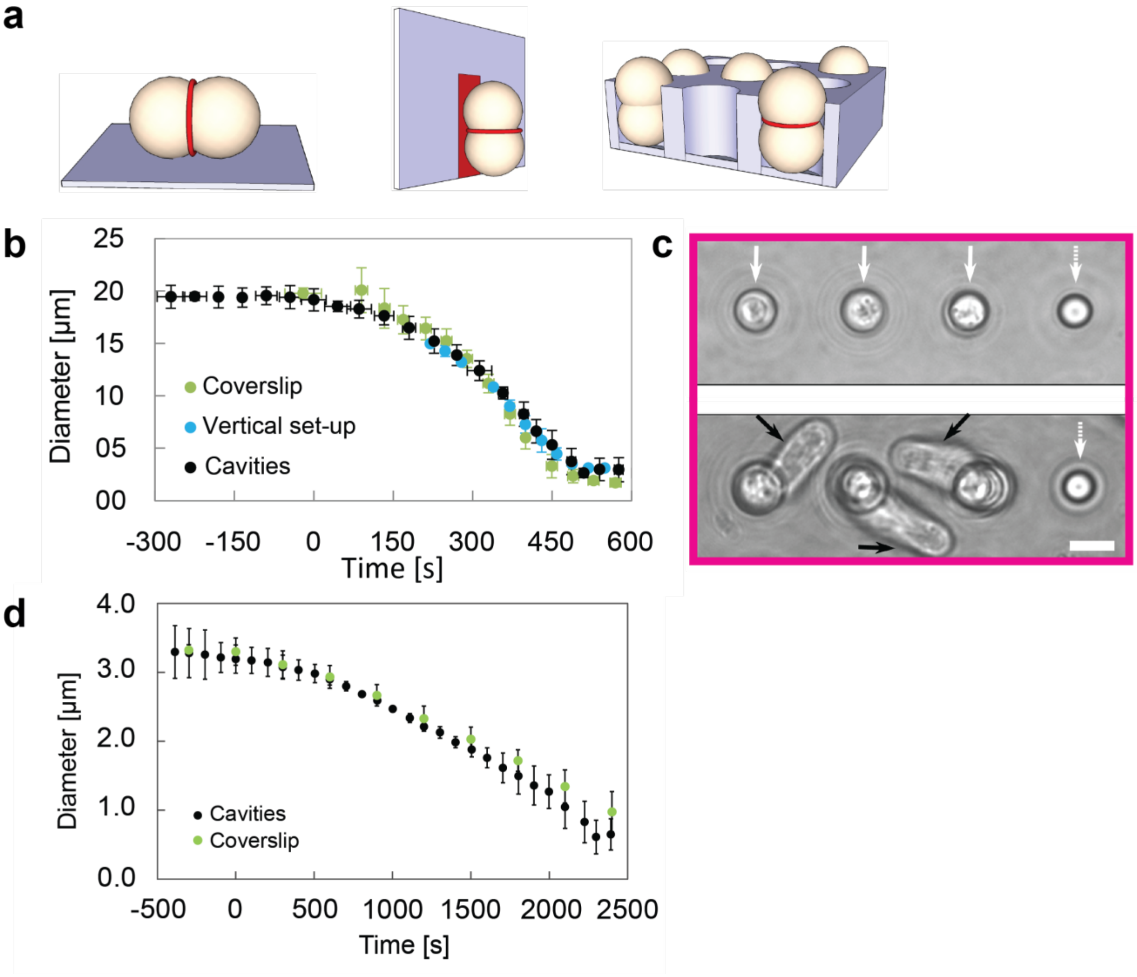

Supplementary Figure 2. Comparison of micro-cavities with standard surfaces

a, Schematic representation of the three set-ups and their comparison. Coverslip: Cell division on untreated glass coverslips, Vertical set-up: Cell division on vertically oriented glass coverslips. The coverslips are patterned with fibronectin lines, Cavities: Cells division in micro-cavities. **b**, Constriction behavior of HeLa cells on coverslips (green), in the vertical setup (blue) and in micro-cavities (black). Error bars indicate the standard deviation, coverslips: N = 12, vertical setup: N = 6, micro-cavities: N = 18. **c**, Fission yeast cells grow normally in wells, white arrows show cavities filled with cells and black arrows show sister cells after ~ 10 h of growth and division (compare top and bottom panels). The dashed arrow points at an empty well[1]. Scale bar, 4 µm. **d,** Constriction behavior of fission yeast cells on coverslips (green) and in micro-cavities (black). Error bars indicate the standard deviation, coverslips: N = 12, micro-cavities: N = 29.

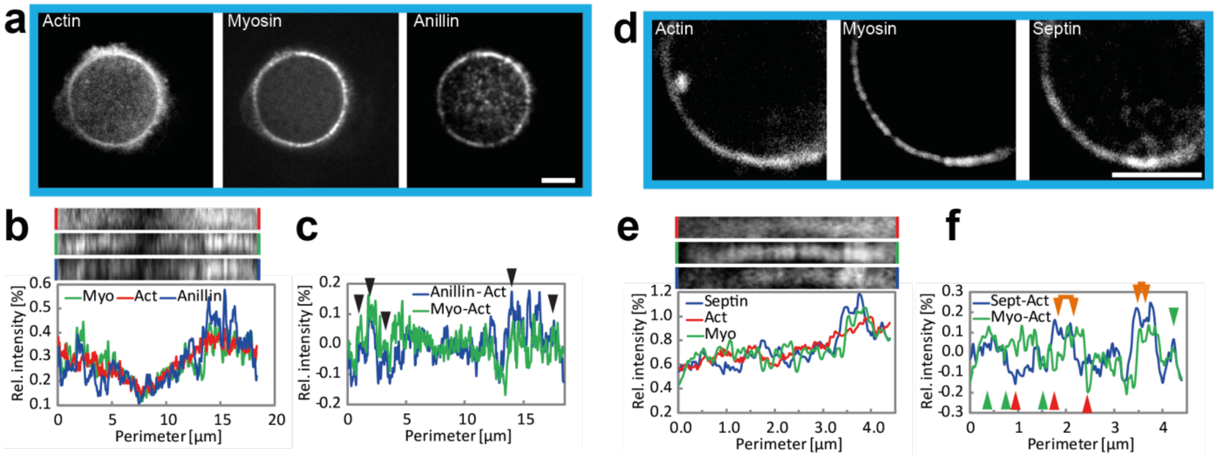

Supplementary Figure 3. Anillin and septin in the cytokinetic ring of mammalian cells

a, HeLa cells expressing LifeAct-mCherry and myosin-GFP stained for anillin. As myosin, anillin shows clusters distribution. Smoothen with ImageJ, scale bar 5 µm. **b**, Polar transformation of the rings and the corresponding intensity spectra. The intensities are normalized by their total intensity. Anillin and myosin profiles show peaks whereas actin spectrum is rather flat. **c**, Anillin and myosin spectra are subtracted by the actin signal, to flatten the profiles. Anillin and myosin show colocalization in some parts (arrows). **d**, HeLa cells expressing LifeAct-mCherry and myosin-GFP stained for septin7. Septin profile reveals clusters. Smoothen with ImageJ, scale bar 5 µm. **e**, Polar transformation of the indicated part of the rings and the corresponding intensity spectra. The intensities are normalized by their total intensity. Septin and myosin show peaks whereas the actin spectrum is rather flat. **f**, Septin and myosin spectra subtracted by the actin signal. Arrows indicate where profiles show colocalization (green), anti-correlation (red) and a shift (orange).

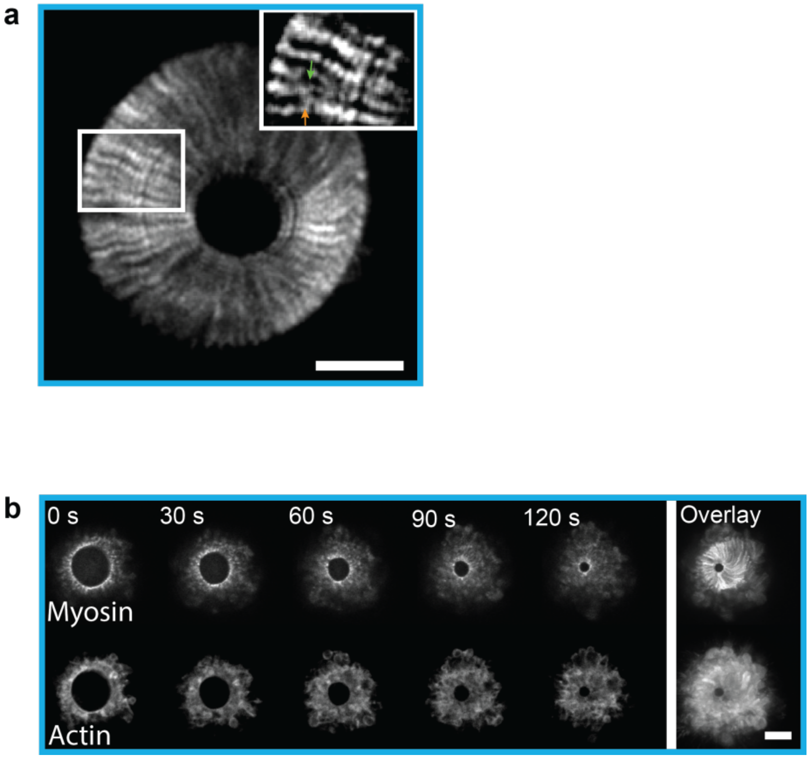

Supplementary Figure 4. Movements of myosin clusters in mammalian cells

a-b, Characteristics of clusters during ring closure. **a,** Overlay of rings in myosin. The clusters undergo fusion. The inset shows cluster fusion (indicated by arrows). Scale bar, 5 µm. **b,** Time lapse series of ring closure in actin and myosin. The overlay of the time series shows the rotation of the patterns/radial path of the clusters during constriction. Scale bar, 5 µm.

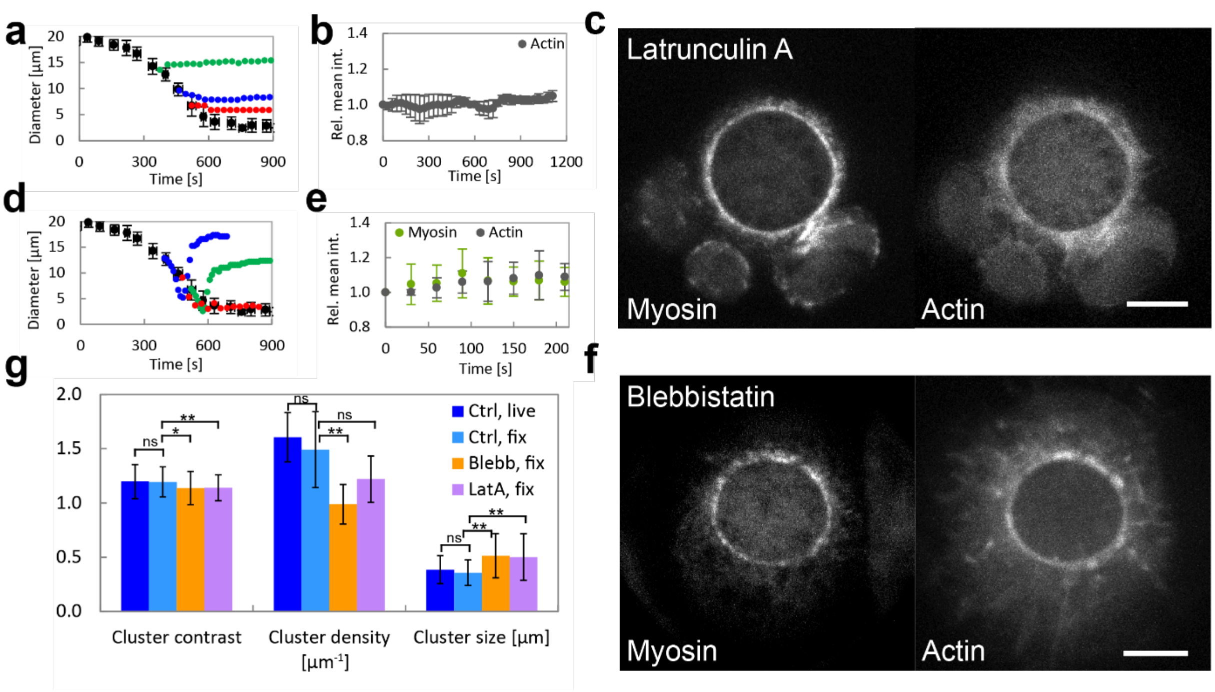

Supplementary Figure 5. Effects of cytoskeleton drugs on the cytokinetic ring in mammalian cells

a, The constriction behaviors of three representative rings in the presence of 100 µM blebbistatin (in black without blebbistatin). Ring closure is stalled upon blebbistatin addition.

b, After incubation with 100 µM blebbistatin the mean intensity of actin remains constant. Error bars indicate the standard deviation, N = 6. **c,f,** Cells fixed after incubation with c) latrunculin A (1.5 µM, 3 min) and f) blebbistatin (100 µM, 15 min). Scale bars 5 µm.

d, Three representative constriction behavior after incubation with 1.5 µM latrunculin A: the ring continues to constrict in some cases (red); and opens in others (blue and green). In all cases the constriction speed is faster after addition of the drug. **e,** With 1.5 µM latrunculin A, myosin and actin concentrations remain constant. Error bars indicate the standard deviation, N = 6. **g**, Mean value of cluster contrast, density and size for cells treated with drugs and fixed and untreated cells (Ctrl, live and fixed). Mean value for rings in the diameter range from 9 µm to 12 µm (Number of rings: N(Ctrl, live) = 16, N(Ctrl, fix) = 8, N(Lat A) = 9, N(Blebb) = 7, number of measurements of cluster contrast and cluster size: between 88 and 211 per conditions, number of measurements of cluster density: between 8 and 16 per condition). Mann-Whitney test was performed, ns not significantly different (P>0.05), * significantly different (P<0.05), ** significantly different (P<0.01). Error bars indicate the standard deviation.

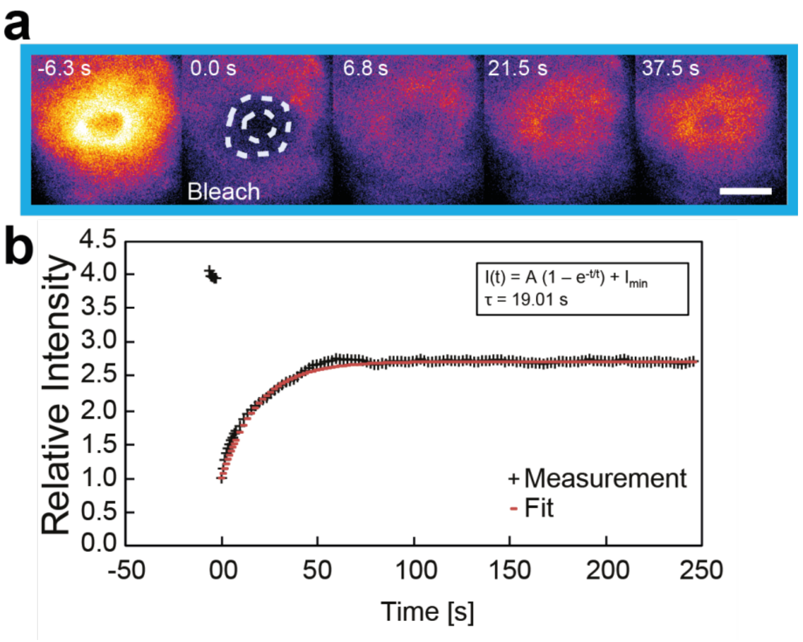

Supplementary Figure 6. Myosin FRAP in mammalian ring
a, FRAP experiment on myosin in the rings of HeLa cells. The entire ring is bleached. Scale bars 5 µm. **b**, The recovery is not complete. Its half time is ~20 s.

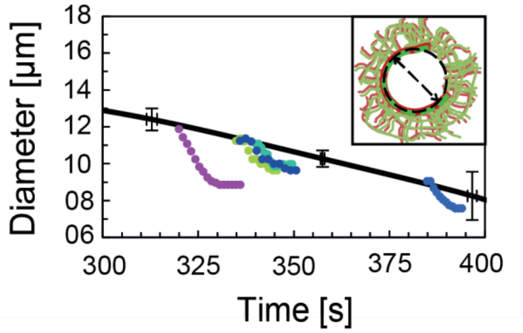

Supplementary Figure 7. Constriction behaviors after ablation of the cytokinetic ring in mammalian cells

Constriction behaviors after ablation of the cytokinetic ring in mammalian cells. The cut rings were fitted with a circle and their diameter measured as a function of time. The individual curves were aligned to the constriction curve of intact rings (Fig. 1e). The cut rings constrict faster than control rings.

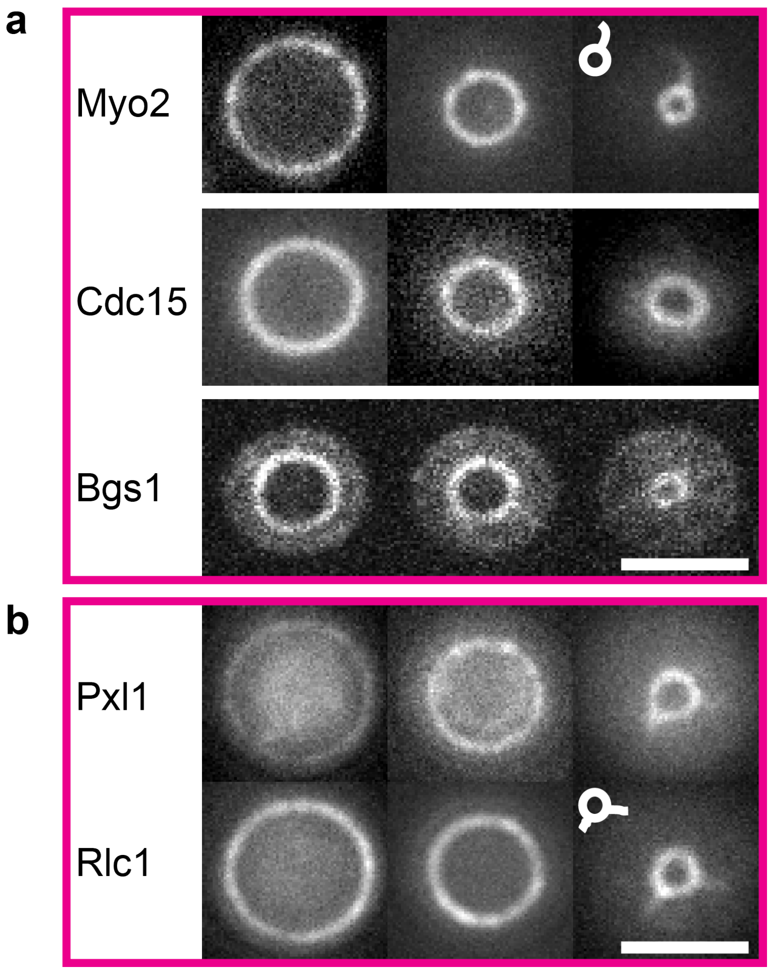

Supplementary Figure 8. Other cytokinetic ring proteins in fission yeast

a–b, Different cytokinetic proteins of fission yeast ring. **a**, Cells labeled with Myo2, Cdc15 and Bgs1. **b**, Pxl1 and Rlc1 on the same ring (top/bottom panels). Note the arms depicted in white. Scale bar, 2 µm.

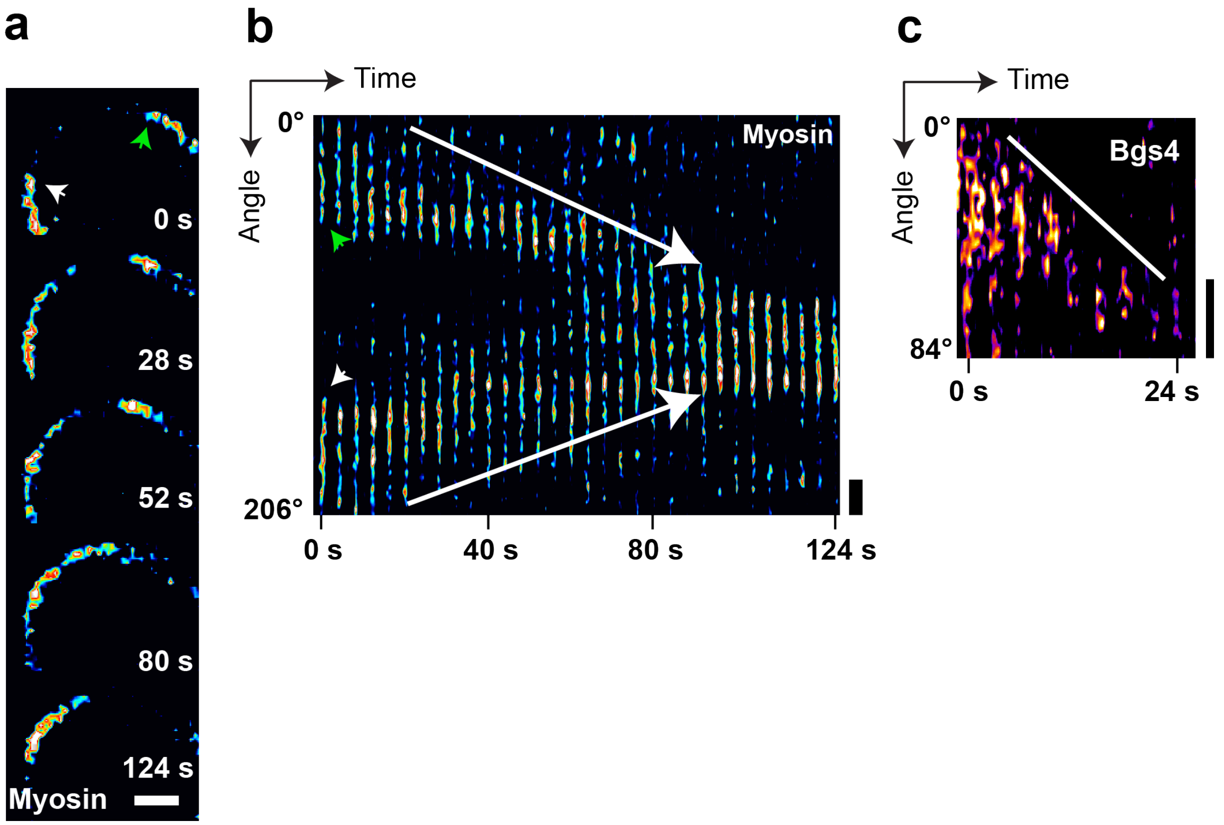

Supplementary Figure 9. Cluster rotations in fission yeast

a-b, Myosin clusters motion in fission yeast (a) and its kymograph after polar transformation (b). Small arrows point to the same clusters in (a) and in (b) at time 0 s, and large arrows in (b) indicate their rotations.

c, Rotation of a Bgs4 cluster. Scale bars, 2 µm.

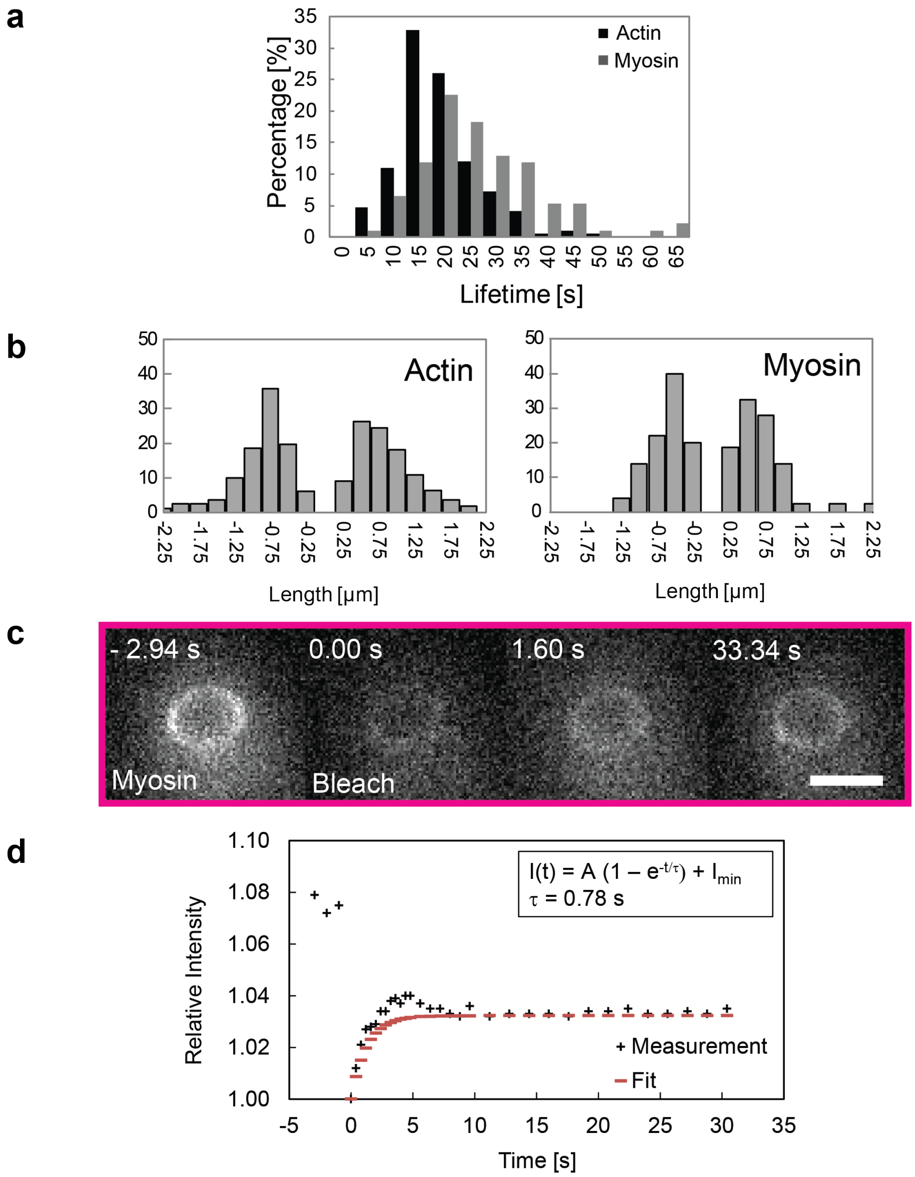

Supplementary Figure 10. Cluster characteristics and myosin FRAP in fission yeast
a–b, Cluster characteristics of fission yeast ring constriction. **a**, Histogram for lifetime of clusters. The mean lifetime is 16.4 s (s.d. = 7.7 s, n= 192) for actin and 24.1 s (s.d. = 11.6 s, n= 93) for myosin. **b,** Distance of cluster movement for cluster rotating clockwise and counterclockwise. The mean distance for all actin cluster is 0.72 µm (s.d. = 0.41 µm, n = 192) and 0.52 µm (s.d. = 0.34 µm, n = 93) for myosin cluster. **c–d,** FRAP experiment on myosin in the fission yeast cytokinetic ring. **c**, The entire ring is bleached, and partially recovers. Scale bar, 2 µm. **d,** The half time of recovery is ~1s.

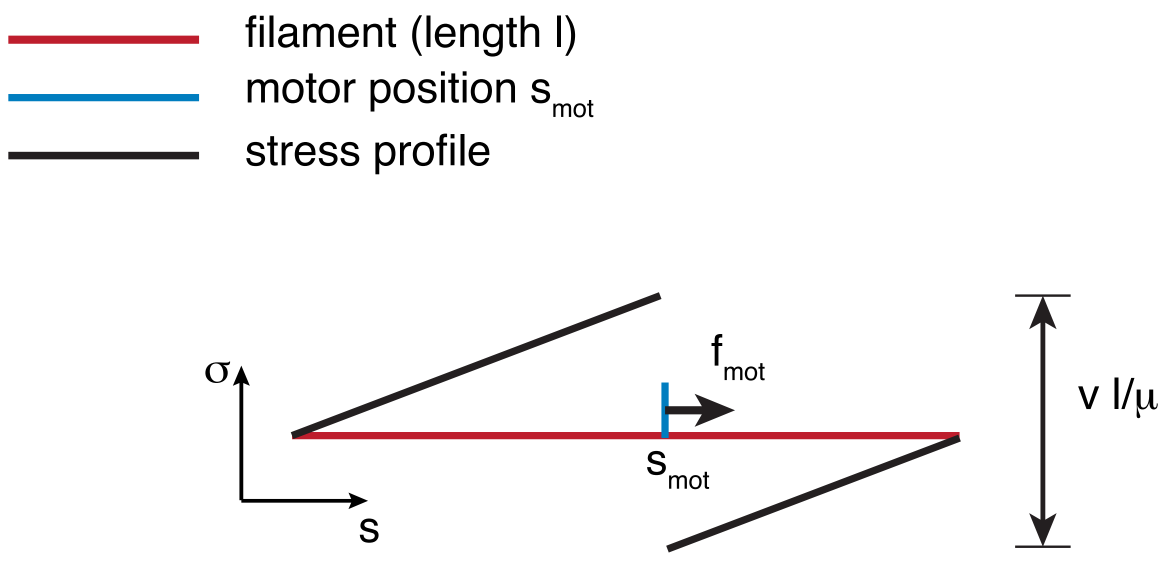

Supplementary Figure 11. Illustration of the stress distribution along a stiff slender rod

The rod is of length ℓ (the filament, red) that is drawn by a motor force f_{mot} at s_{mot} into the direction of the arrow. The filament velocity is v, its mobility μ. The black line indicates the stress profile that results from the applied force and the filament friction with the environment.

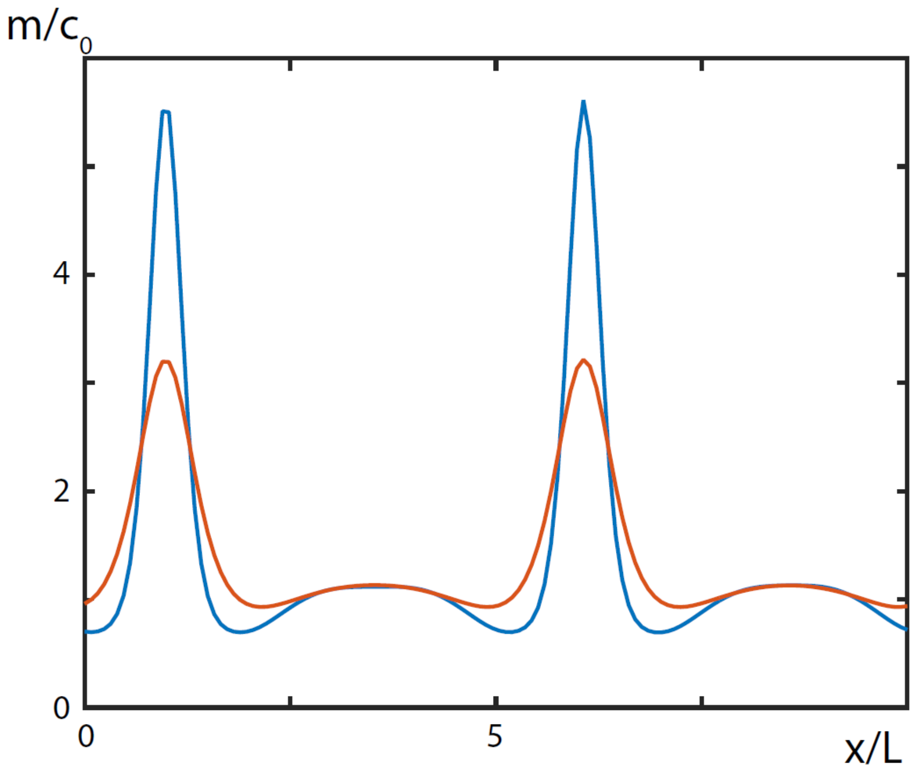

Supplementary Figure 12. Motor distribution m for two values of the motor activity α

Blue: $\alpha = 4\ell\omega_d$, orange: $\alpha = 2.5\ell\omega_d$ mimicking the effect of adding blebbistatin. Other parameters: perimeter $L = 10\ell$, $\beta = 0.1\ell\omega_d$, $\omega_d = 0.2$, $\omega_c = 1$, $v = 2.5\ell\omega_d c_0$, $D = \ell^2\omega_d c_0$. The total filament number was $\int dx\{2c_{bp}(x) + c^+(x) + c^-(x)\} = 2Lc_0$.

Supplementary Table

Supplementary Table 1: Strain genotypes, markers and sources.

Strain	Genotype	Fluorescent markers	Source / Reference
DR 2	pxl1::kanMX6 leu1+::GFPpxl1+ rlc1-mCherry::natR	Rlc1-mCherry myosin II regulatory light chain GFP-Pxl1 paxillin-like protein	This study
JW 1110	h^+ kanMX6-Pmyo2-mYFP-myo2 ade6-M210 leu1-32 ura4-D18	Myo2-YFP myosin II heavy chain	Wu and Pollard, 2005
PPG 5054	h^+ pxl1::kanMX6 leu1+::GFPpxl1+ leu1-32 ura4-D18	GFP-Pxl1 paxillin-like protein Pxl1	Pinar et al., 2008
JW 977	h^+ cdc15-mYFP-kanMX6 ade6-M210 leu1-32 ura4-D18	Cdc15-YFP membrane-cytoskeletal interactions, F-BAR domains	Wu and Pollard, 2005
519	h^- bgs1Δ::ura4+ P_{bgs1}+::GFP-bgs1+:leu1+ leu1-32 ura4-D18 his3- Δ1	GFP-Bgs1 1,3-beta-glucan synthase catalytic subunit Bgs1	Cortes et al., 2002
561	h^- bgs4Δ::ura4+ P_{bgs4}+::GFP-bgs4+:leu1+ leu1-32 ura4-D18 his3-Δ1	GFP-Bgs4 1,3-beta-glucan synthase catalytic subunit Bgs4	Pilar Pérez
JW1348	h^+ nmt41-GFP-CHD (rng2)-leu1$^+$ rlc1-mCherry-natMX6 ade6-M210 leu1-32 ura4-D18	Rlc1-mCherry myosin II regulatory light chain CHD-GFP calponin homology domain	Vavylonis et al., 2008
JW1349	h^+ nmt41-GFP-CHD (rng2)-leu1+ rlc1-tdTomato natMX6 ade6-M210 leu1-32 ura4-D18	Rlc1-tdTomato myosin II regulatory light chain CHD-GFP calponin homology domain	Vavylonis et al., 2008
JM207	h^+ rlc1-mCherry::natR ade- leu- ura-	Rlc1-mCherry myosin II regulatory light chain	James B. Moseley
PN 4461	h^- cps1-191 rlc1-GFP::KanR	Rlc1-mCherry myosin II regulatory light chain	Nurse lab. collection

Supplementary Note 1

MEAN-FIELD MODEL FOR THE DYNAMICS OF ACTIN FILAMENTS IN THE CONTRACTILE RING

In the following, we will give the mathematical details of the physical model used in the main text.

Model definition

With our theory, we try to capture essential features of the ring dynamics, such as, filament polarity, rules of interaction between filaments through molecular motors. Consequently, the final equations of motion describe the behavior of contractile rings independently of many details of the molecular interaction rules. Still, in the following, we will evoke a specific image to introduce the dynamic equations.

Consider a ring of perimeter L of actin filaments such that the filaments align with the ring perimeter. We denote the co-ordinate along the ring perimeter by x and describe the distribution of (polar) actin filaments along x by the densities c^{+} for filaments with their plus-end pointing clockwise and c^{-} for filaments of the opposite orientation. Two filaments of opposite orientation can join their plus-ends forming a bipolar filament (Fig. 4a, (i)). Indirect evidence for such a process is given by the fusion of nodes observed in fission yeast[2]. While we refrain from suggesting an explicit molecular mechanism, such bipolar filaments may be formed by motor clusters linking the filaments. Also actin nucleating proteins of the formin family could be involved (Figs. 2c and 3b). The distribution of bipolar filaments is denoted by c_{bp} and gives the density of their centers. Bipolar filaments form at rate $\omega_c c^{+} c^{-}$, bipolar filaments can split into two filaments of opposite orientations at rate ω_d (Fig. 4a, (i)).

Actin filaments continuously turn over. In general, they assemble at the barbed end by addition of actin monomers and disassemble at the pointed end by actin monomer removal or severing. Assembly and disassembly can be captured by effective rates. These rates depend on the state of the ends: Capping proteins can inhibit or promote assembly and disassembly. In a minimal model of the ring dynamics, we refrain from giving a detailed account of filament assembly and disassembly. Instead we assume that all filaments have a fixed length that is equal to the average filament length ℓ. Bipolar filaments thus have a length of 2ℓ and the total actin density at a point x is $\int_0^{\ell} d\xi \left(c^{+}(x+\xi) + c_{bp}(x+\xi) + c^{-}(x-\xi) + c_{bp}(x-\xi)\right)$. As a crude account of filament turnover, we will assume that filaments assemble and disassemble at the two ends at the same

rate. This leads to an apparent motion of the polar filaments at velocity $\pm v_{to}$.

Let us now turn to the filament dynamics induced by molecular motors. They can induce relative sliding between actin filaments. The corresponding velocities are α between filaments of the same orientations (Fig. 4a, (ii)) and β for filaments of opposite orientations (Fig. 4a, (iii)). We use these parameters to quantify the strength of the motor-mediated filament- filament interactions. We assume that motors are located at the filaments' plus-ends, such that $c^+ + c^- + c_{bp}$ is the distribution of motors. Finally, fluctuations are accounted for by diffusion terms with an effective diffusion constant D. The corresponding dynamic equations read:

$$
\begin{aligned}
\partial_t c^+(x) = {} & D\partial_x^2 c^+(x) - \partial_x \alpha \int_0^\ell d\xi \left(c^+(x+\xi) - c^+(x-\xi)\right) c^+(x) \\
& - \partial_x \alpha \int_0^\ell d\xi \, c_{bp}(x+\xi) c^+(x) + \partial_x \beta \int_0^\ell d\xi \left(c^-(x-\xi) + c_{bp}(x-\xi)\right) c^+(x) \\
& - \partial_x v_{to} c^+(x) - \omega_c c^+(x) c^-(x) + \omega_d c_{bp}(x)
\end{aligned}
$$

$$
\begin{aligned}
\partial_t c^-(x) = {} & D\partial_x^2 c^-(x) - \partial_x \alpha \int_0^\ell d\xi \left(c^-(x+\xi) - c^-(x-\xi)\right) c^-(x) \\
& + \partial_x \alpha \int_0^\ell d\xi \, c_{bp}(x-\xi) c^-(x) - \partial_x \beta \int_0^\ell d\xi \left(c^+(x+\xi) + c_{bp}(x+\xi)\right) c^-(x) \\
& + \partial_x v_{to} c^-(x) - \omega_c c^+(x) c^-(x) + \omega_d c_{bp}(x)
\end{aligned}
$$

$$
\begin{aligned}
\partial_t c_{bp}(x) = {} & D\partial_x^2 c_{bp}(x) - \partial_x \alpha \int_0^\ell d\xi \left(c_{bp}(x+\xi) - c_{bp}(x-\xi)\right) c_{bp}(x) \\
& - \partial_x \alpha \int_0^\ell d\xi \left(c^-(x+\xi) - c^+(x-\xi)\right) c_{bp}(x) \\
& - \partial_x \beta \int_0^\ell d\xi \left(c^+(x+\xi) - c^-(x-\xi)\right) c_{bp}(x) + \omega_c c^+(x) c^-(x) - \omega_d c_{bp}(x)
\end{aligned}
$$

To assist the reader, let us state explicitly the difference of this model to the one discussed in Ref.[3], where the framework used here was developed. The present model extends the former work by including the presence of bipolar filaments and processes of their assembly and disassembly. Furthermore, in the original formulation[3], filament assembly and disassembly were neglected. Here, we include it in an effective way, by adding the treadmilling currents. That treadmilling is an important part of the actin assembly dynamics was shown, for example, in Ref.[4].

For numerical solution of the dynamic equations, we used a first-order upwind scheme with adaptive time stepping. For the calculation, we have used dimensionless parameters, where time

has been scaled by $(\omega_c c_0)^{-1}$, length by ℓ, and the filament densities by c_0, where $c_0 L$ is the number of plus- and of minus-filaments. Consequently, v_{to} is scaled by $\ell\omega_c c_0$, α and β by $\ell\omega_c$, and D by $\ell^2\omega_c c_0$.

Mechanism of the instability

First note that the interaction of two bipolar filaments with each other tends to align their centers. For a homogenous ring of bipolar filaments, the force on each bipolar filament cancels out. As soon as there is a perturbation, locally imbalances are present that will lead to an accumulation of bipolar filaments, possibly at different positions along the ring, unless diffusion is dominating and smoothing the perturbations. What is the typical distance one can expect between two clusters of bipolar filaments? A bipolar filament can interact with all bipolar filaments that are a distance ℓ away. These bipolar filaments extend a distance 2ℓ from the original filament's center, which suggests that the typical distance between two clusters is about 4ℓ. This is indeed the typical distance we observe after clusters have developed starting from a random perturbation of the homogenous state. The typical distance is also affected by the system size (the ring perimeter). For $L = 10\ell$, which we use in the main text, only two clusters are seen. Note that the distances between clusters changes on long time scales, which presumably eventually leads to a single remaining cluster for systems of any size. However, this coarsening process takes place on such long time scales that it is irrelevant for the dynamics of contractile rings and not further discussed here.

Calculation of the stress in the bundle

The stress in the bundle is defined as the sum of the stresses in the individual filaments[3]. Stresses in a filament are generated by motors that pull on the filaments and by friction with the surrounding medium. Explicitly, force balance on a single filament gives

$$\partial_s \sigma = \frac{1}{\mu} v + f_{mot}.$$

In this expression, s is the co-ordinate along the filament, σ the stress in the filament, μ a mobility, v the filament's velocity, and f_{mot} the force density exerted by motors on the filament. Only the effects of motors cross-linking two filaments are accounted for. The stress along a filament is thus piece-wise linear in s with slope v/μ, where $v = \pm\alpha$ or $v = \pm\beta$ depending on the orientation and the relative position of the partner filament, the motor is connected to. If there is no motor at a filament end, then the stress vanishes at this point, and the stress jumps by an amount $|v|\ell/\mu$ at the positions s_{mot}, where motors are bound to the filament, see Fig. S11. The

total stress profile along the bundle is then obtained by summing the stress profiles along all filaments in the bundle. Since the expressions are quite involved, we refrain from giving them here explicitly.

Behavior after addition of blebbistatin

To capture the effect of the myosin inhibitor blebbistatin in our model, we reduced the motor activity α, compared to a case leading to a stationary state that corresponds to the pattern in mammalian rings. For stationary states, the myosin clusters subsequently broadened, see Fig. S12. This qualitatively agrees with the behavior observed in mammalian rings, see Fig. 2d in the main text. Similar results have been obtained when instead of α the values of ℓ and v_{to} were reduced to mimic the effects of latrunculin A.

Supplementary Methods

Analysis of rings

For mammalian cells, we measured the total and mean fluorescence intensities by tracing the ring contour with ImageJ. The total and mean intensity were normalized by the total and mean intensity of the cells at the onset of division (t = 0 s). The normalized values were averaged and the standard deviation is given by the error bars (Fig. 1 f, g). With intensities extracted from live samples, we measured the bleaching rate of the cytoplasmic pool of fluorescent proteins, as a good indicator for bleaching since the recovery times for FRAP at the ring were within seconds. The results yielded minor corrections for intensity measurements, within 10%. In addition, fixed samples gave the same measures as live samples, showing that corrections for photobleaching were not needed.

Fission yeast rings can be fitted by a circle. Therefore, we measured the intensities by measuring the intensity in circles of the dimensions of the outer ring diameter and the inner ring diameter with ImageJ. The subtraction of these two values gave the total fluorescence intensity of the ring. By dividing the total intensity by the area of the ring (which is the area of the outer circle minus the inner circle), we calculated the mean intensity. Since the variations in intensity measures between individual cells are small in fission yeast cells, we took snapshots of individual cells and assigned time points to the rings as a function of their diameter. The averaged intensity curve was then normalized with respect to the intensity value of 3.1 μm. For intensity measurements of rings before constriction where the diameter is constant we analyzed timelapse movies. We normalized the intensity with respect to the intensity at a diameter of 3.1 μm. Measurements on snapshots and timelapses are in agreement and they are plotted in Fig. 1 i, j (timelapse data until 250 s, then data from fixed cells).The standard deviation is given by the error bars (Fig. 1 i, j).

Microfabrication

Polydimethylsiloxane (PDMS) was mixed with its curing agent (Sylgard 184, Dow Corning) in a 10:1 ratio, and the solution allowed to degas for 30 min. A porous membrane filter (pore diameter 5.0 μm, shiny face up, Millipore Isopore, TMTP01300) was placed at the bottom of a

Petri dish and the sticky side of Scotch tape was applied to the top of the filter[1]. The tape with the filter was attached to a double sided tape attached to a Petri dish with the filter side exposed to the air, the PDMS poured onto the filters (non-shiny face up), and the mixture allowed to cure overnight, followed by 4 h curing at 65°C before the stamp was peeled off.

Alternatively the stamp can be fabricated by means of microfabrication (Supplementary Fig. 1) We used regular arrays of microcavities surfaces prepared using standard lithographic methods on silicon wafers[5]. Circular patterns on a mask can be transferred to a Si-Wafer. The surface will contain holes of the size of the wells. PDMS is mixed with curing agent (10:1) and poured on the wafer. Air bubbles are removed by degassing for 30 min. After 4 h at 65°C the PDMS will be cured and the stamp can carefully be cut out and peeled of the wafer.

The stamp was exposed to a plasma cleaning for 1 min (Harrick Plasma, PDC-32G, high setting power), followed by a 10 min exposure to Chlorotrimethylsilane 97% (Sigma-Aldrich, C72854, TMCS) vapor or by the deposition of an anti-adhesive layer (Sigma-Aldrich, SL2 Sigmacote).

The liquid degassed PDMS mixture was spread on a glass coverslip #0 (25 mm in diameter, Fisherbrand) with a Pasteur pipette[5,6], after its cleaning with a 1 min exposure in the plasma cleaner. The silanized stamp was then placed onto the PDMS coated coverslip, allowed to cure at room temperature overnight, followed by four hours curing at 65°C. The stamp was separated from the coverslips, generating the well pattern on the upper layer of the 30 µm thick elastomer, using the coverslip as the sealed bottom of the chamber. The overall thickness of the sample allowed objectives with small working distances and high numerical apertures to be used.

We modified the protocol for larger cavities for mammalian cells. The PDMS stamps were activated with a plasma cleaner and silanized with TMCS as described above. Liquid PDMS was spin-coated on the molds at 1500 rpm for 30 s. The PDMS was cured for at least 2 h at 65°C. For plasma binding of the cured PDMS layer to a coverslip (#0), both – the PDMS stamp and the coverslip – were plasma activated. The thin PDMS layer was then pressed on the coverslip. The pressure was maintained for several seconds. After about 30 min the PDMS stamp was unpeeled and the thin PDMS layer containing the microcavities was plasma bound to the coverslip.

Supplementary References

1 Riveline, D. & Buguin, A. Devices and methods for observing the cell division. WO/2010/092116 (2009).

2 Vavylonis, D., Wu, J. Q., Hao, S., O'Shaughnessy, B. & Pollard, T. D. Assembly mechanism of the contractile ring for cytokinesis by fission yeast. *Science* **319**, 97-100 (2008).

3 Kruse, K. & Julicher, F. Self-organization and mechanical properties of active filament bundles. *Phys Rev E Stat Nonlin Soft Matter Phys* **67**, 051913 (2003).

4 Erlenkamper, C. & Kruse, K. Treadmilling and length distributions of active polar filaments. *J Chem Phys* **139**, 164907 (2013).

5 Balaban, N. Q. *et al.* Force and focal adhesion assembly: a close relationship studied using elastic micropatterned substrates. *Nat Cell Biol* **3**, 466-472 (2001).

6 Riveline, D. *et al.* Focal contacts as mechanosensors: externally applied local mechanical force induces growth of focal contacts by an mDia1-dependent and ROCK-independent mechanism. *J Cell Biol* **153**, 1175-1186 (2001).
